# Whole-Genome Sequence of Listeria monocytogenes Serovar 4b Strain IZSAM_Lm_hs2008, Isolated from a Human Infection in Italy

**DOI:** 10.1128/genomeA.00053-15

**Published:** 2015-03-05

**Authors:** Patrizia Centorame, Vicdalia Aniela Acciari, Massimiliano Orsini, Marina Torresi, Luigi Iannetti, Andrea Angius, Dante Di Giammartino, Vincenza Annunziata Prencipe, Giacomo Migliorati

**Affiliations:** a National Reference Laboratory for *Listeria monocytogenes*, Istituto Zooprofilattico Sperimentale dell’Abruzzo e del Molise G. Caporale, Teramo, Italy; b Center for Advanced Studies, Research and Development in Sardinia (CRS4), Loc Piscina Manna, Pula, Cagliari, Italy; c Infectious Diseases Unit, Ospedale Civile G. Mazzini, Teramo, Italy

## Abstract

Listeria monocytogenes is one of the most important foodborne pathogens. In this report, we present the complete and annotated genome of L. monocytogenes sequence type 06 (ST06) serovar 4b strain IZSAM_Lm_hs2008, isolated from an adult immunocompetent patient who developed the disease and died.

## GENOME ANNOUNCEMENT

Listeria monocytogenes is a facultative intracellular bacterium that is widespread in the environment and responsible for infections occurring through the ingestion of contaminated food. Not all strains of L. monocytogenes are equally capable of causing disease in humans. Isolates belonging to serovar 4b are the major organisms responsible for outbreaks and sporadic cases of human listeriosis, suggesting that this serovar may possess unique virulence properties (1). The genome of L. monocytogenes is composed of one circular chromosome with or without plasmids.

In this study, we characterized the L. monocytogenes strain IZSAM_Lm_hs2008, isolated from an adult male resident in Italy not belonging to any particular high-risk category. He showed typical symptoms of fever, altered mental status, rigor nuchalis, and lethal exitus. The complete genome of this strain was determined with the Illumina HiSeq 2000 platform (100-bp paired-end sequencing library; insertion size, 300 bp; coverage, >200×). Quality control, trimming, assembly, and preliminary genome annotation were carried out under the Orione framework (2) using the SPAdes software (3) for assembly, while finishing was completed using the DraftDoctor software (version 1.0 CRS4; M. Orsini [https://code.google.com/p/draftdoctor/]). Genome annotation was manually curated after a preliminary annotation was performed by Prokka (4). This strain was further characterized by pulsed-field gel electrophoresis (PFGE) (AscI and ApaI enzymes) and multilocus sequence typing (MLST). PFGE yielded a macrorestriction combined pattern that has not been observed since 2003 in food samples collected in Italy, while multilocus sequence typing (MLST) (http://www.pasteur.fr/mlst) revealed that the isolate belongs to sequence type 06 (ST06) grouped into clonal complex 6 (CC6). The genome of the L. monocytogens IZSAM_Lm_hs2008 strain consists of a circular chromosome of 2,904,228 bp, with a G+C content of 38%. There are 2,805 coding sequences (CDSs), which represent 88.6% of the genome (average length, 918 nucleotides [nt]), 7 full rRNA operons, and 67 tRNAs. No plasmids or prophages were detected. The availability of the genome sequence from this strain can provide insight into genetic mechanisms of pathogenicity, especially if compared to other clinical L. monocytogenes strains.

### Nucleotide sequence accession number.

The complete genome of this isolate has been deposited in GenBank with the accession no. CP010346.
